# LINC00665/miRNAs axis-mediated collagen type XI alpha 1 correlates with immune infiltration and malignant phenotypes in lung adenocarcinoma

**DOI:** 10.1515/med-2022-0478

**Published:** 2022-07-13

**Authors:** Jun Zhu, Yuan Weng, Fudong Wang, Jun Zhao

**Affiliations:** Department of Thoracic Surgery, The First Affiliated Hospital of Soochow University, Medical College of Soochow University, Suzhou 215006, Jiangsu Province, China; Department of Thoracic Surgery, The First Affiliated Hospital of Soochow University, Medical College of Soochow University, No. 899 Pinghai Road, Gusu District, Suzhou 215006, Jiangsu Province, China; Institute of Thoracic Surgery, The First Affiliated Hospital of Soochow University, No. 899 Pinghai Road, Gusu District, Suzhou 215006, Jiangsu Province, China; Department of Thoracic Surgery, Affiliated Hospital of Jiangnan University, Wuxi 214062, Jiangsu Province, China

**Keywords:** lung adenocarcinoma, COL11A1, long non-coding RNA, prognosis, immune infiltration

## Abstract

Collagen type XI alpha 1 (COL11A1) as an oncogene has been reported in several malignant tumors. Herein, we aimed to explore the function of COL11A1 and its upstream regulators in lung adenocarcinoma (LUAD). COL11A1 expression prognostic significance, gene ontology, Kyoto Encyclopedia of Genes and Genomes, and immune infiltration were explored in LUAD. *In vitro* experimental measurements were implemented to validate the function of COL11A1 and LINC00665 in LUAD cells. Our study demonstrated that LINC00665-2 and COL11A1 were significantly upregulated in LUAD tissues compared with nontumor tissues. COL11A1 was positively correlated with multiple immune cell enrichment, suggesting that COL11A1 may be a prospective therapeutic target to enhance the efficacy of immunotherapy in LUAD. A regulatory mechanism LINC00665-2/microRNAs (miRNAs)/COL11A1 axis was identified to facilitate the tumorigenesis of LUAD. si-LINC00665 transfection induced the inhibition of growth and migration, and apoptosis was reversed by the overexpression of COL11A1 in LUAD cells. In conclusion, LINC00665 as a competing endogenous RNA sponging multiple miRNAs to modulate COL11A1 expression in LUAD, suggesting that LINC00665/miRNAs/COL11A1 axis may contribute to the pathogenesis of LUAD.

## 1 Introduction

Collagen type XI alpha 1 (COL11A1) is a component of XI collagen and is primarily existed in the cartilage [1]. Importantly, emerging evidence suggests that COL11A1 is a cancer progression-correlated gene that can facilitate tumor growth, migration, invasion, metastasis, and chemotherapy resistance [2]. In addition, upregulation of COL11A1 is associated with cancer recurrence and poor survival and serves as a diagnostic indicator [3,4,5,6]. Several reports also validate that COL11A1 is an oncogene in the progression of non-small cell lung cancer (NSCLC) [7,8]. Herein, we aimed to further investigate the upstream regulator of COL11A1 in lung adenocarcinoma (LUAD).

Numerous long noncoding RNAs (lncRNAs) have been authenticated to play important biological functions in physiological and pathological processes [9,10]. Classically, lncRNAs function as competing endogenous RNAs (ceRNAs) to sponge microRNAs (miRNAs), which are a class of small non-coding RNAs as post-transcriptional repressors to degrade messenger RNA (mRNA) or reduce protein translation via completely or partially binding with the 3′-untranslated regions (3′-UTRs) of mRNA [11,12,13,14], which has massively focused on metastasis, tumor microenvironment and immune infiltration of LUAD [15,16,17]. Aberrantly expressed lncRNAs, such as lncRNA JPX, LINC00312, LINC00673-v4 and TMPO-AS1, have been implicated in the initiation and progression of LUAD [18,19,20,21]. LINC00665 is upregulated and functions as an oncogene in multiple malignant tumors, including breast cancer, hepatocellular carcinoma, and endometrial carcinoma [22,23,24]. The oncogenic capability of LINC00665 is also identified as a ceRNA via modulating miRNA and its gene target in LUAD [25,26,27,28,29].

In our study, COL11A1 expression was significantly upregulated in LUAD tissues using the GEPIA database (http://gepia.cancer-pku.cn/index.html). We also found that nine miRNAs (hsa-let-7a-5p, hsa-let-7b-5p, hsa-let-7c-5p, hsa-let-7d-5p, hsa-let-7e-5p, hsa-let-7f-5p, hsa-let-7g-5p, hsa-miR-144-3p and hsa-miR-26a-5p) were downregulated in LUAD tissues and might be potential regulators of COL11A1. Previous studies have revealed that several miRNAs, including miR-20a-3p [30], miR-335 [31] and miR-25-3p [32], mediate COL11A1 expression in pancreatic ductal adenocarcinoma, ovarian cancer and renal cancer, respectively. Next, based on the ENCORI database (http://starbase.sysu.edu.cn/), LINC00665 was predicted as a coregulator of nine miRNAs. Furthermore, the biological functions of LINC00665/miRNAs/COL11A1 were validated in LUAD cells and nude-mouse transplanted tumor model.

## 2 Materials and methods

### 2.1 TCGA data analysis

The expression of COL11A1 in LUAD tissues and para-carcinoma in TCGA database was evaluated using ggplot2 package (version 3.3.3) as described previously [33]. Nomogram and calibration curve were analyzed using R software (version 3.6.3) with rms package (version 6.2-0) and survival package (version 3.2-10) as described previously [34].

### 2.2 Acquirement of data from GEPIA

The expression of COL11A1 in LUAD tissues and corresponding nontumor tissues was excavated using the GEPIA platform (http://gepia.cancer-pku.cn/detail.php) [35]. The expression of COL11A1 tumor tissues was matched with TCGA normal and GTEx datasets.

### 2.3 GSEA, GO and KEGG pathway enrichment

Analysis of single gene difference of COL11A1 in LUAD was prepared for GSEA using TCGA database with DESeq2 package (version 1.26.0) as described previously [36]. Top 50 COL11A1-related genes were filtrated using TCGA database with stat package (version 3.6.3). In addition, gene ontology (GO), including biological process (BP), cellular component (CC) and molecular function (MF), and Kyoto Encyclopedia of Genes and Genomes (KEGG) pathways enrichment analysis were predicted by DAVID online database (https://david.ncifcrf.gov/).

### 2.4 Immune infiltration

The correlation of COL11A1 with immune cells was analyzed by GSVA package (version 1.34.0) with ssGSEA algorithm [37,38].

### 2.5 COL11A1-related miRNAs and lncRNAs prediction and analysis

miRNAs and lncRNA were predicted by TargetScan (http://www.targetscan.org/vert_72/) and ENCORI database [39] with the parameter setting as follows: CLIP and Degradome data with high stringency, pan-cancer, program number and predicted program more than 1. Moreover, the expression of KCNQ1OT1, NEAT1 and LINC00665 was analyzed using the GEPIA platform (http://gepia.cancer-pku.cn/detail.php) [35]. The expression of miRNAs in LUAD was calculated using R software (version 3.6.3) and ggplot2 package (version 3.3.3) based on the TCGA database.

### 2.6 Cell culture and transfection

16HBE, H1975 and A549 cells were cultured as described previously [40]. LncRNA LINC00665 siRNAs and COL11A1 overexpressed plasmids were obtained from Gene-Pharma (Shanghai, China), and transfected as suggested in the previous study [40].

### 2.7 Reverse transcription-quantitative polymerase chain reaction and western blot

Reverse transcription–polymerase chain reaction and western blot were performed as described previously [40,41]. Anti-COL11A1 primary antibody (ab64883; dilution: 1:1,000; Abcam; Cambridge, UK) was used to incubate protein membranes.

### 2.8 Cell proliferation, migration and apoptosis

Cell proliferation and migration were implemented as our previous study [41]. Cell apoptosis was analyzed using a commercial TUNEL kit (Roche).

### 2.9 Statistical analysis

The Shapiro-Wilk normality test, unpaired and paired tests with Mann-Whitney *U* test and Wilcoxon signed-rank test, log-rank test, univariate Cox regression analysis and spearman analysis were implemented to evaluate the significant difference with *P* < 0.05.


**Ethics approval and consent to participate:** Not applicable.

## 3 Results

### 3.1 COL11A1 expression in Pan-cancer

**Figure 1:**
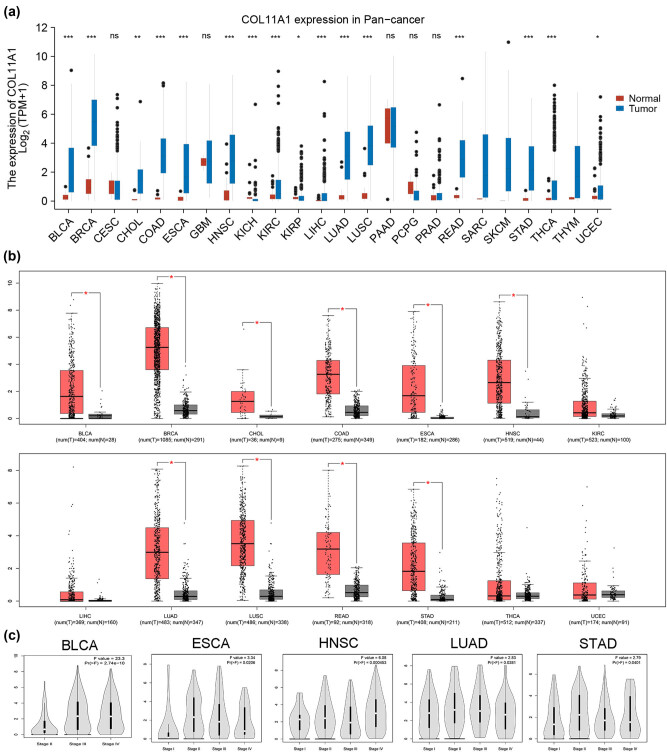
COL11A1 expression in Pan-cancer. COL11A1 expression in 24 cancer types was evaluated using the TCGA database (a). GEPIA database was utilized to validate COL11A1 expression in 14 cancer types (b). GEPIA database for the detection of COL11A1 in BLCA, ESCA, HNSC, LUAD and STAD with different stages (c). ^*^ *P* < 0.05, ^**^ *P* < 0.01 and ^***^ *P* < 0.001 compared with the control group.

COL11A1 expression in 24 cancer types was evaluated using the TCGA database. As shown in Figure 1a, COL11A1 was significantly increased in 14 cancer types (BLCA, BRCA, CHOL, COAD, ESCA, HNSC, KIRC, LIHC, LUAD, LUSC, READ, STAD, THCA and UCEC) and decreased in 2 cancer types (KICH and KIRP) compared with corresponding normal tissues. GEPIA database was utilized to validate COL11A1 expression in 14 cancer types. Our results demonstrated that COL11A1 was significantly elevated in 10 cancer types (BLCA, BRCA, CHOL, COAD, ESCA, HNSC, LUAD, LUSC, READ and STAD) compared with the corresponding normal tissues (Figure 1b). GEPIA database also revealed that COL11A1 was significantly correlated with cancer stage in BLCA, ESCA, HNSC, LUAD and STAD (Figure 1c).

### 3.2 COL11A1 correlates with OS in LUAD

**Figure 2:**
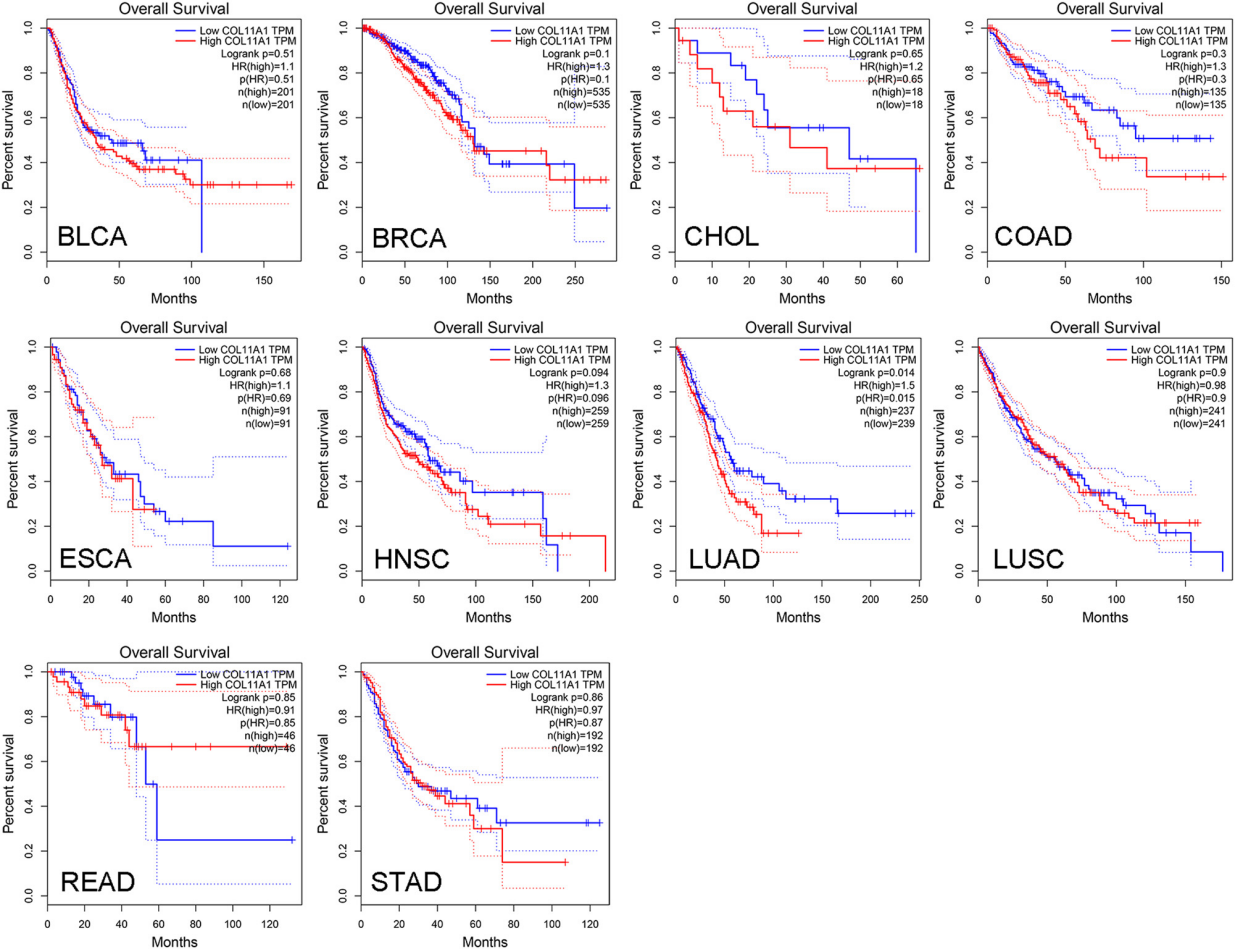
COL11A1 correlates with overall survival (OS) in LUAD. The association between COL11A1 and OS in 10 cancer types was determined by the GEPIA database.

The association between COL11A1 and OS in 10 cancer types was determined by the GEPIA database. COL11A1 high expression in LUAD exhibited an unfavorable prognosis. However, no significant prognostic value of COL11A1 was observed in other cancer types (Figure 2).

### 3.3 COL11A1 as a prognostic and diagnostic indicator in LUAD

**Figure 3:**
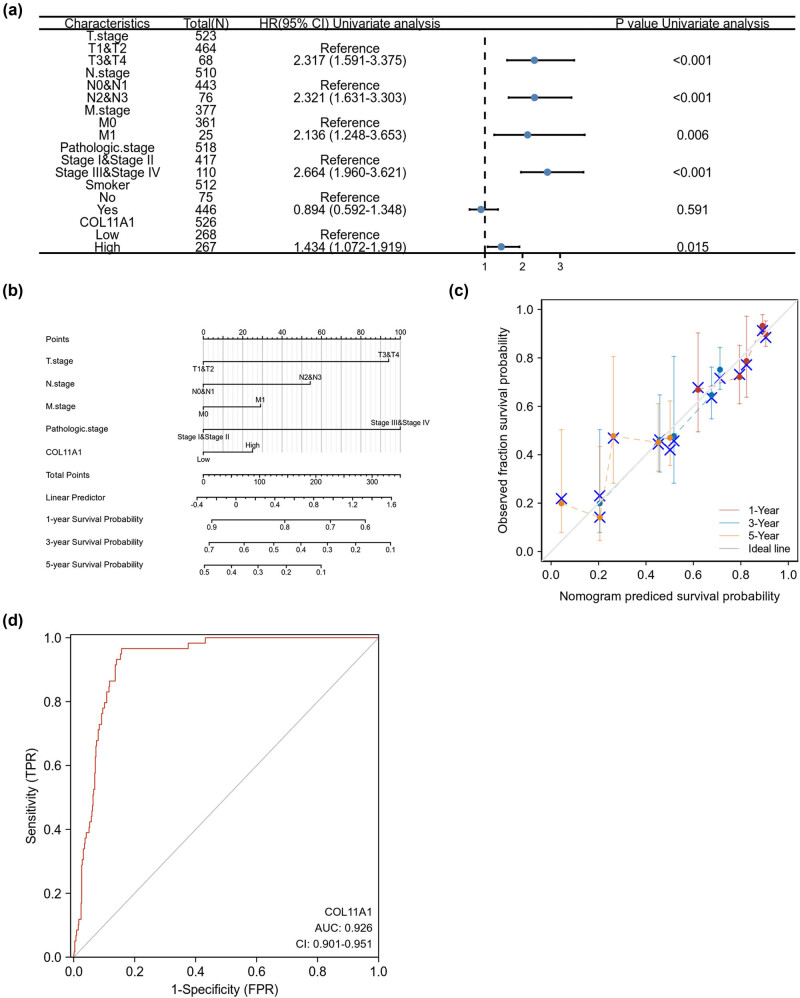
COL11A1 as a prognostic and diagnostic indicator in LUAD. Univariate Cox regression analysis for OS-related risk factors in LUAD patients (a). A nomogram represented that a higher total point was correlated with a worse survival probability (b). The calibration curve validated that nomogram might be a better model for predicting OS in patients with LUAD (c). The diagnostic significance of COL11A1 was evaluated by ROC curve with AUC using TCGA database (d).

As mentioned earlier, COL11A1 high expression was correlated with a poor OS of patients with LUAD. We also performed univariate COX regression analysis to evaluate OS-related risk factors in LUAD patients. As shown in Figure 3a, poor T (hazard ratio (HR) = 2.317; 95% confidence interval (CI): 1.591–3.375; *P* < 0.001), N (HR = 2.321; 95% CI: 1.631–3.303; *P* < 0.001), M (HR = 2.136; 95% CI: 1.248–3.653; *P* = 0.006) and pathologic stage (HR = 2.664; 95% CI: 1.960–3.621; *P* <0.001) and high COL11A1 (HR = 1.434; 95% CI: 1.072–1.919; *P* = 0.015) expression were unfavorable prognostic factors of OS for LUAD patients. In addition, we established a prediction model of OS by fitting with clinical parameters (T, N, M and pathologic stage) and COL11A1 expression based on the TCGA database. As shown in Figure 3b, the nomogram represented that a higher total point was correlated with a worse survival probability. The calibration curve validated that nomogram might be a better model for predicting OS in patients with LUAD (Figure 3c). We also evaluated the diagnostic significance of COL11A1 using the TCGA database. As shown in Figure 3d, COL11A1 (area under the curve (AUC) = 0.926; 95% CI: 0.901–0.951; sensitivity = 0.966; specificity = 0.843) might be a favorable diagnostic biomarker to distinguish LUAD from normal subjects.

### 3.4 GSEA, GO and KEGG pathway enrichment analysis

**Figure 4:**
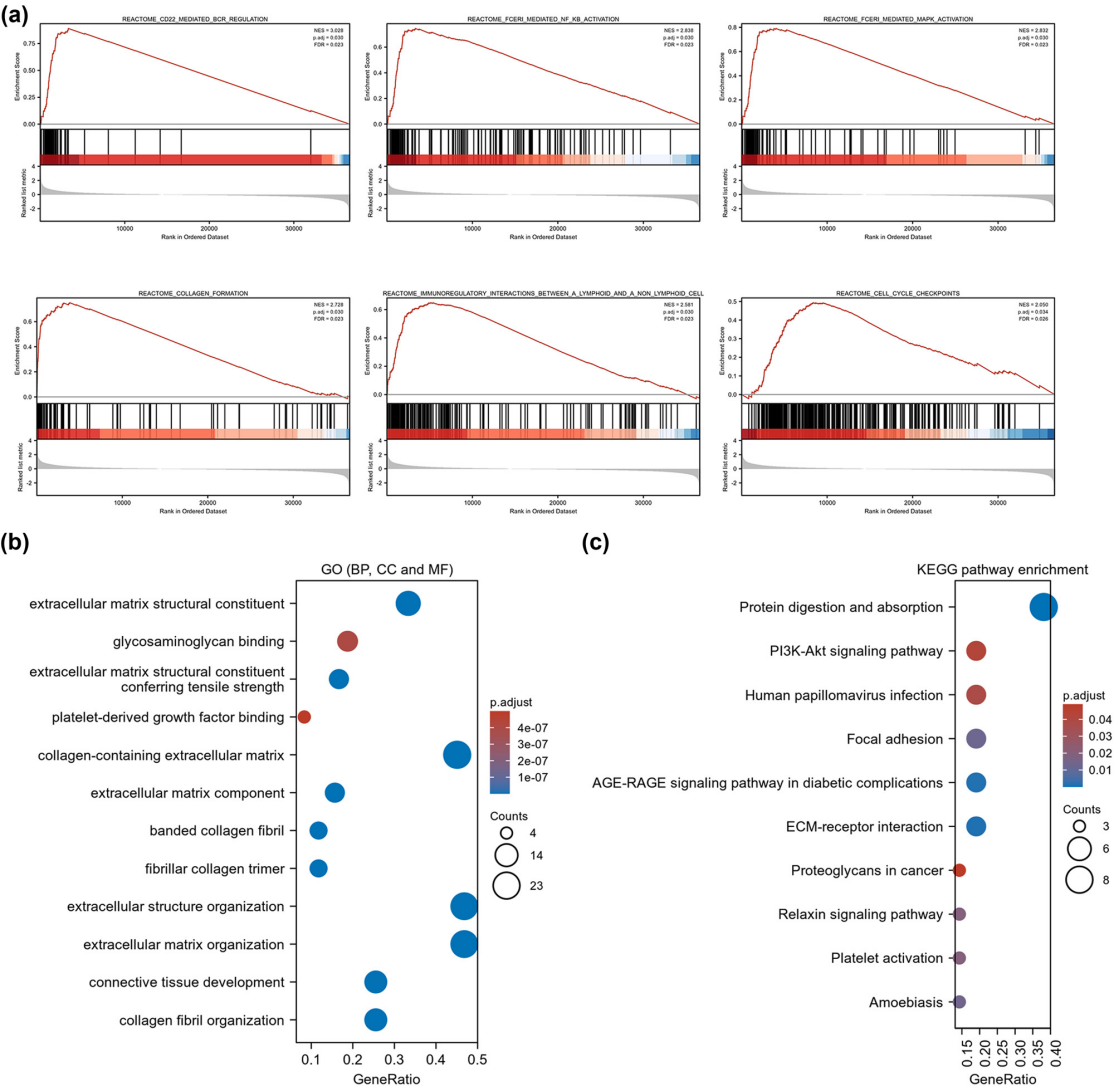
Assessment of GSEA, GO and KEGG in LUAD. GSEA was implemented using the TCGA database (a). GO (b) and KEGG (c) were executed based on the top 50 related genes.

To further analyze the biological functions of COL11A1 in LUAD, GSEA was implemented using the TCGA database. Top six terms were listed as follows (Figure 4a and Table S1): CD22-mediated B cell receptor regulation (normalized enrichment score (NES) = 3.028, *P* = 0.030), Fc epsilon receptor (FCERI)-mediated nuclear factor-kappa B (NF-κB) activation (NES = 2.838, *P* = 0.030), FCERI-mediated mitogen-activated protein kinase (MAPK) activation (NES = 2.832, *P* = 0.030), collagen formation (NES = 2.728, *P* = 0.030), immunoregulatory interactions between a lymphoid and a non-lymphoid cell (NES = 2.581, *P* = 0.030) and cell cycle checkpoints (NES = 2.050, *P* = 0.034). These findings suggested that COL11A1-related genes were enriched in tumor-associated signaling pathways NF-κB and MAPK activation, cell growth, collagen formation and immunoregulatory activity. Next, GO and KEGG pathway enrichment analyses were executed based on the top 50 related genes. One hundred thirty-two terms of BP, 9 terms of CC and 32 terms of MF were significantly enriched by the GO analysis, and the top four terms of BP, CC and MF were listed as shown in Figure 4b. As shown in Figure 4c, the top 10 terms of pathways included, protein digestion and absorption, extracellular matrix (ECM)-receptor interaction, AGE-RAGE signaling pathway in diabetic complications, focal adhesion, amoebiasis, platelet activation, relaxin signaling pathway, human papillomavirus infection, PI3K-Akt signaling pathway and proteoglycans in cancer. KEGG enrichment analysis revealed that COL11A1-related genes were correlated with protein digestion and absorption, ECM-receptor interaction and focal adhesion. These pathways have been validated to accelerate cell migration and invasion in lung cancer [42,43].

### 3.5 COL11A1 correlates with immune infiltration in LUAD

**Figure 5:**
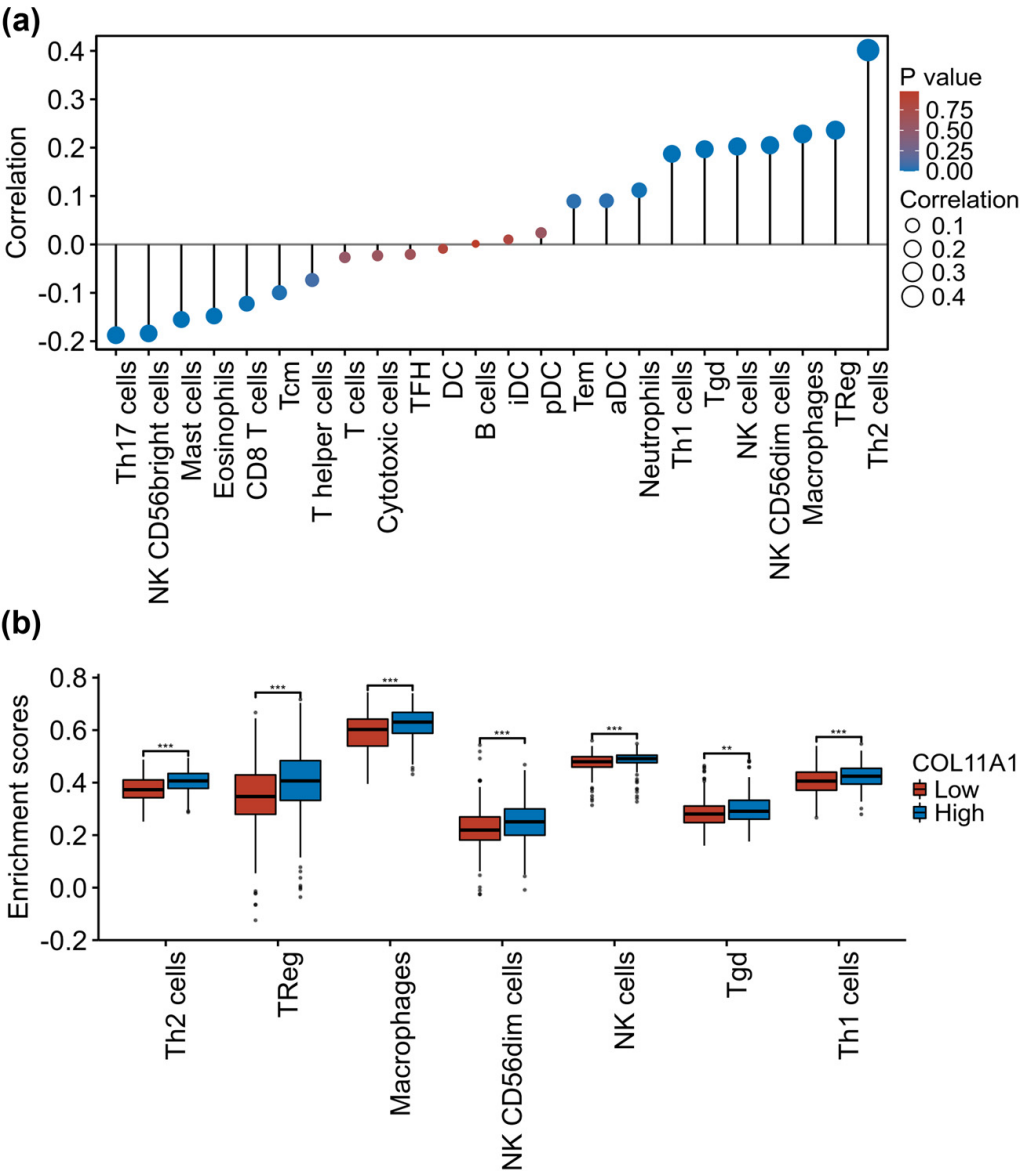
COL11A1 correlates with immune infiltration in LUAD. ssGSEA algorithm was used to evaluate the correlation between COL11A1 and immune cell enrichment in LUAD (a). The enrichment score of seven immune cells, including Th2 cells, TReg, macrophages, NK CD56dim cells, NK cells, Tgd and Th1 cells, was evaluated between COL11A1 high expression group and low expression group (b).

As mentioned earlier, COL11A1-related genes were significantly enriched in CD22-mediated B cell receptor regulation and immunoregulatory interactions between a lymphoid and a nonlymphoid cell. These findings suggested that COL11A1 may be associated with tumor immune response. Herein, ssGSEA algorithm was used to evaluate the correlation between COL11A1 and immune cell enrichment in LUAD. As shown in Figure 5a and Table 1, COL11A1 was significantly positively correlated with 10 immune cells (Th2 cells, regulatory T (Treg), macrophages, natural killer (NK) CD56dim cells, NK cells, Tgd, Th1 cells, Neutrophils, aDC and Tem) enrichment and negatively correlated with six immune cells (Th17 cells, NK CD56bright cells, mast cells, eosinophils, Tcm and CD8 T cells) enrichment in LUAD. In addition, we found that a higher enrichment score of seven immune cells, including Th2 cells, TReg, macrophages, NK CD56dim cells, NK cells, Tgd and Th1 cells, was observed in COL11A1 high expression group than those of in the COL11A1 low expression group (Figure 5b).

**Table 1:** Relationship between COL11A1 and immune cell enrichment in LUAD

Molecule	Immune cell	Spearman correlation	*P* value
COL11A1	Th2 cells	0.402	<0.001
COL11A1	TReg	0.236	<0.001
COL11A1	Macrophages	0.228	<0.001
COL11A1	NK CD56dim cells	0.205	<0.001
COL11A1	NK cells	0.202	<0.001
COL11A1	Tgd	0.197	<0.001
COL11A1	Th17 cells	−0.188	<0.001
COL11A1	Th1 cells	0.187	<0.001
COL11A1	NK CD56bright cells	−0.184	<0.001
COL11A1	Mast cells	−0.155	<0.001
COL11A1	Eosinophils	−0.148	<0.001
COL11A1	CD8 T cells	−0.122	0.005
COL11A1	Neutrophils	0.112	0.010
COL11A1	Tcm	−0.100	0.021
COL11A1	aDC	0.090	0.037
COL11A1	Tem	0.089	0.039
COL11A1	T helper cells	−0.073	0.090
COL11A1	T cells	−0.027	0.537
COL11A1	pDC	0.024	0.580
COL11A1	Cytotoxic cells	−0.023	0.592
COL11A1	TFH	−0.021	0.636
COL11A1	iDC	0.010	0.813
COL11A1	DC	−0.009	0.835
COL11A1	B cells	0.001	0.976

### 3.6 Prognostic significance of COL11A1 expression based on immune cell enrichment in LUAD patients

**Figure 6:**
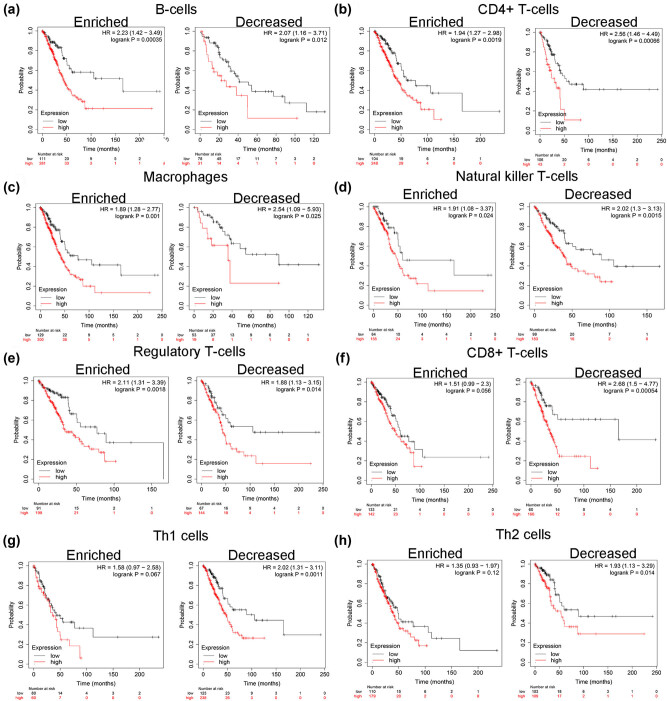
Prognostic significance of COL11A1 expression based on immune cell enrichment in LUAD patients. The association between COL11A1 and OS was analyzed in enriched and decreased immune cell subgroups of B cells (a), CD4+ T cells (b), CD8+ T cells (c), macrophages (d), NK T-cells (e), Treg cells (f), Th1 cells (g) and Th2 cells (h).

As mentioned earlier, COL11A1 was correlated with poor prognosis and immune cell enrichment in LUAD. Therefore, we hypothesize whether COL11A1-regulated poor prognosis was associated with immune cells enrichment. The association between COL11A1 and OS was analyzed in enriched and decreased immune cell subgroups. In both enriched and decreased subgroups of B cells (Figure 6a), CD4+ T cells (Figure 6b), macrophages (Figure 6c), NK T-cells (Figure 6d) and Treg cells (Figure 6e), COL11A1 high expression was significantly correlated with poor OS in LUAD patients. However, COL11A1 high expression correlated poor OS was observed in LUAD patients with the decrease of CD8+ T cells (Figure 6f), Th1 (Figure 6g) and Th2 cells (Figure 6h), suggesting that COL11A1-related poor OS may be partially mediated by the reduction of CD8+ T cells, Th1 and Th2 cells.

### 3.7 COL11A1-related miRNAs

**Figure 7:**
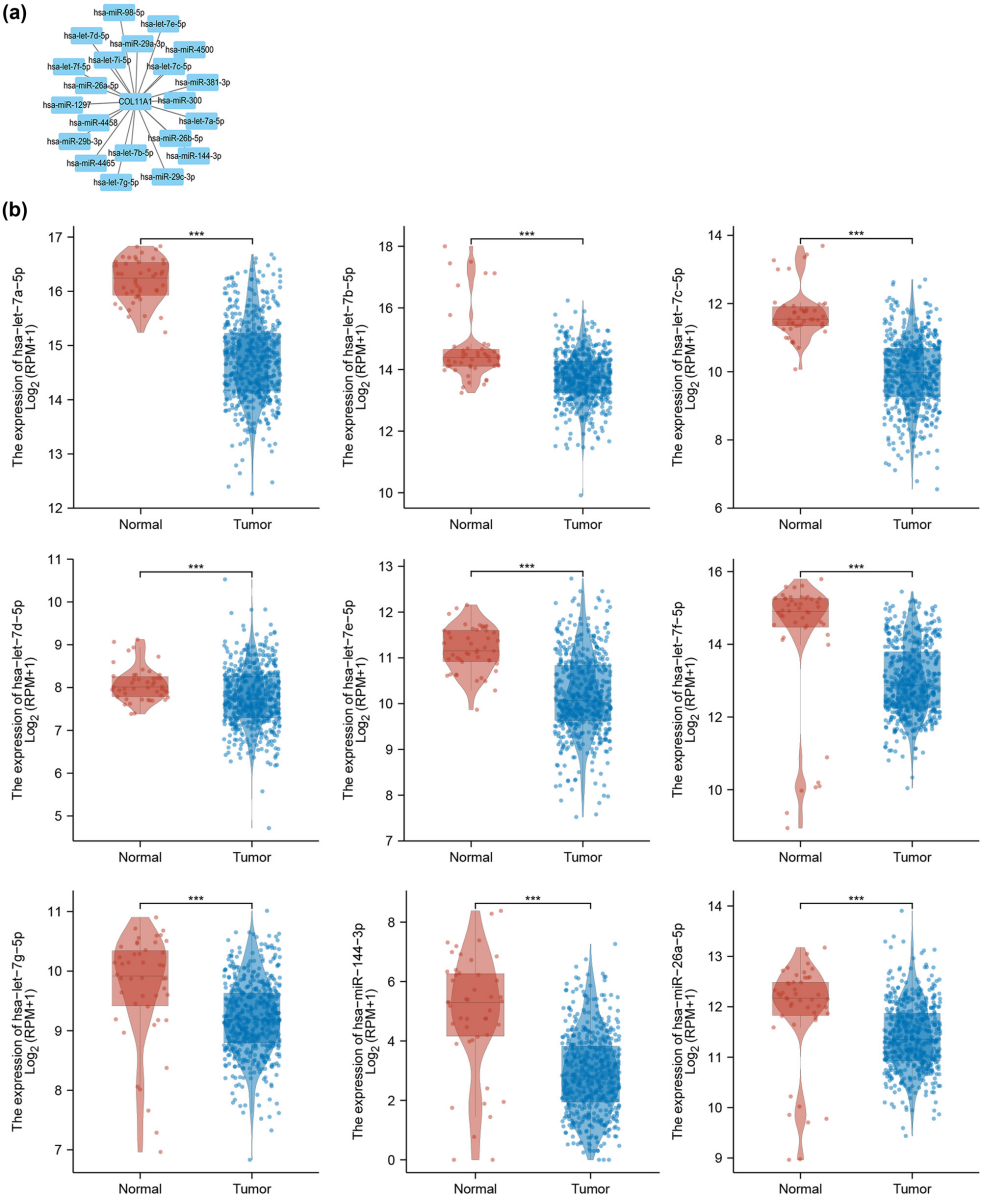
COL11A1-related miRNAs. TargetScan online database was used to predict the potential miRNAs regulators of COL11A1 (a). A total of nine miRNAs (hsa-let-7a-5p, hsa-let-7b-5p, hsa-let-7c-5p, hsa-let-7d-5p, hsa-let-7e-5p, hsa-let-7f-5p, hsa-let-7g-5p, hsa-miR-144-3p and hsa-miR-26a-5p) expression were significantly downregulated in LUAD tissues compared with normal tissues in TCGA database (b). ^***^ *P* < 0.001 compared with control group.

To explore the posttranscriptional repressors, well known as miRNAs, of COL11A1, TargetScan online database was used to predict the potential miRNAs regulators of COL11A1. A total of 21 miRNAs were filtrated as potential regulators of COL11A1 that could bind with 21 miRNAs at conserved sites in the 3′-UTR (Figure 7a). As shown in Figure 7b and Table 2, a total of nine miRNAs (hsa-let-7a-5p, hsa-let-7b-5p, hsa-let-7c-5p, hsa-let-7d-5p, hsa-let-7e-5p, hsa-let-7f-5p, hsa-let-7g-5p, hsa-miR-144-3p and hsa-miR-26a-5p) expression were significantly downregulated in LUAD tissues compared with normal tissues in TCGA database.

**Table 2:** COL11A1-related miRNAs

			Spearman correlation	
Gene	miRNA	Log2FC	*r*	*P*
COL11A1	hsa-let-7a-5p	−1.466	−0.19	<0.001
COL11A1	hsa-let-7b-5p	−0.927	−0.11	0.017
COL11A1	hsa-let-7c-5p	−1.761	−0.18	<0.001
COL11A1	hsa-let-7d-5p	−0.312	−0.16	<0.001
COL11A1	hsa-let-7e-5p	−0.763	−0.063	0.159
COL11A1	hsa-let-7f-5p	−1.198	−0.2	<0.001
COL11A1	hsa-let-7g-5p	−0.506	−0.25	<0.001
COL11A1	hsa-miR-144-3p	−2.021	−0.15	0.001
COL11A1	hsa-miR-26a-5p	−0.478	−0.23	<0.001

### 3.8 LINC00665 mediates COL11A1 expression in LUAD cells

**Figure 8:**
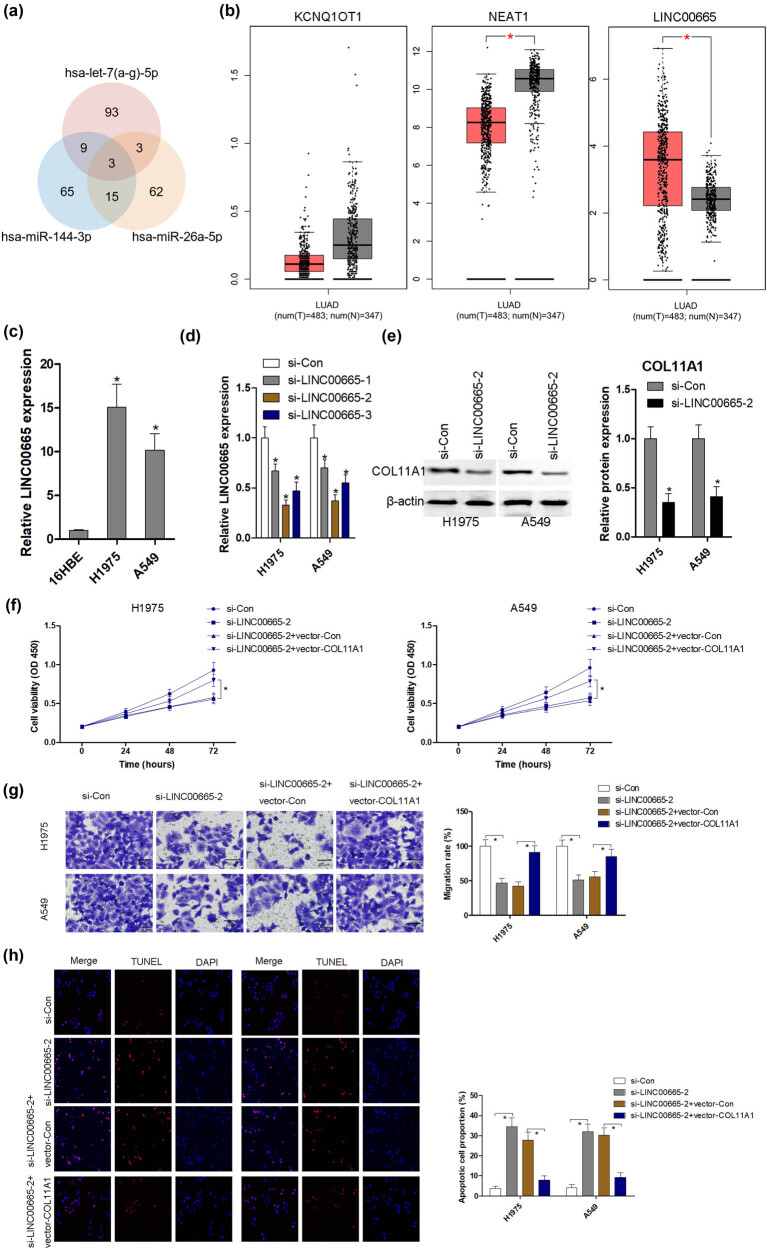
LINC00665 mediates COL11A1 expression in LUAD cells. The upstream lncRNAs of miRNAs was also predicted using the ENCORI database (http://starbase.sysu.edu.cn/) (a). The expression of three lncRNAs (KCNQ1OT1, NEAT1 and LINC00665) in LUAD tissues were analyzed using the GEPIA database (b). The expression level of LINC00665 was measured in H1975 and A549 LUAD cell lines (c). After transfection with three different siRNAs into H1975 and A549 cells, the expression level of LINC00665 was measured using RT-qPCR (d). After transfection with si-LINC00665, the protein expression of COL11A1 was measured using western blot (e). After transfection with si-LINC00665, cell proliferation, migration and apoptosis were measured using CCK-8 (f), transwell (g; magnification 400×; scale bar = 50 μm) and TUNEL (h) assays. ^*^ *P* < 0.05 compared with control group.

**Figure A1:**
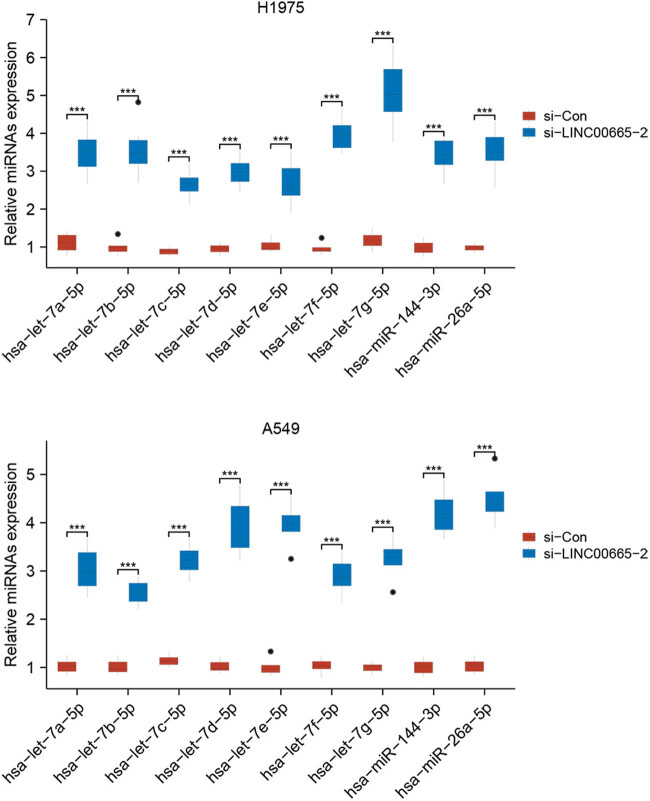
LINC00665 regulates miRNAs expression in LUAD cells. After transfection with si-Con or si-LINC00665 into H1957 and A549 cells, 9 miRNAs (hsa-let-7a-5p, hsa-let-7b-5p, hsa-let-7c-5p, hsa-let-7d-5p, hsa-let-7e-5p, hsa-let-7f-5p, hsa-let-7g-5p, hsa-miR-144-3p, hsa-miR-26a-5p) expression were measured using RT-PCR. ^***^P < 0.001 compared with control group.

**Figure A2:**
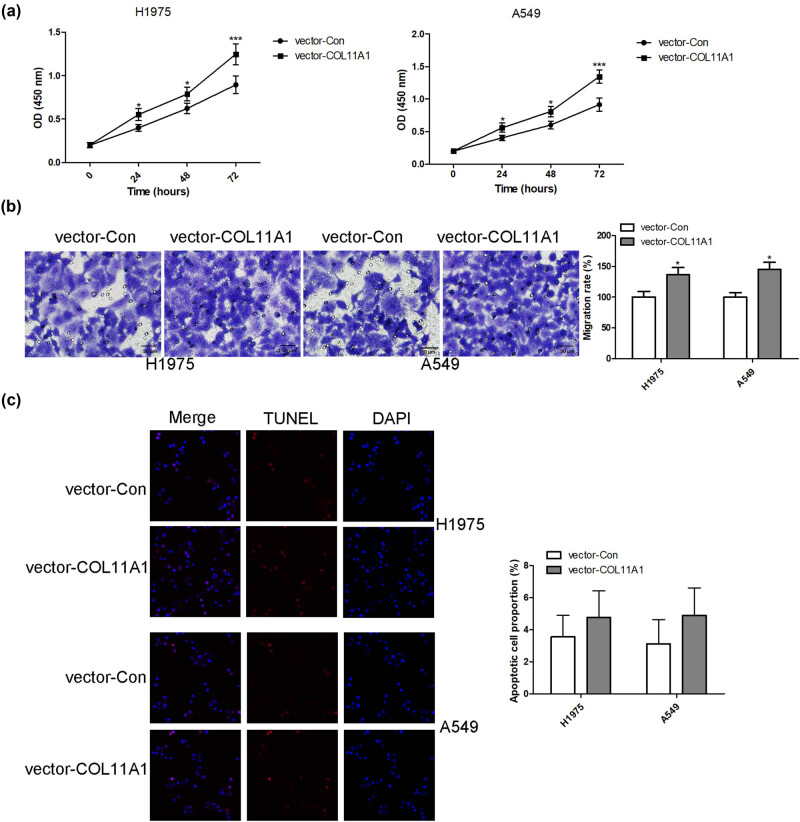
Overexpression of COL11A1 promotes malignant phenotypes of LUAD cells. After transfection with vector-Con or vector-COL11A1 into H1957 and A549 cells, cell growth, migration and apoptosis were evaluated by CCK8 (a), transwell (b) and TUNEL (c) assays, respectively. ^*^P < 0.05 compared with control group.

The upstream lncRNAs of miRNAs were also predicted using the ENCORI database (http://starbase.sysu.edu.cn/). As shown in Figures 8a, three potential lncRNAs (KCNQ1OT1, NEAT1 and LINC00665) were authenticated as the regulators of nine miRNAs. Next, the expression levels of three lncRNAs in LUAD tissues were analyzed using the GEPIA database. The expression of KCNQ1OT1 had no obvious difference between LUAD tissues and nontumor tissues (Figure 8b). However, NEAT1 was significantly downregulated, and LINC00665 was significantly upregulated in LUAD tissues compared with nontumor tissues (Figure 8b). To investigate the role of LINC00665 in the progression of LUAD, the expression level of LINC00665 was measured in H1975 and A549 LUAD cell lines. Compared with normal pulmonary epithelial cells 16HBE, the LINC00665 expression level was significantly elevated in LUAD cell lines H1975 and A549 (Figure 8c). As shown in Figure 8d, transfection with three different siRNAs into H1975 and A549 cells, a significant decrease in LINC00665 expression level was observed. In addition, the expression levels of nine miRNAs (hsa-let-7a-5p, hsa-let-7b-5p, hsa-let-7c-5p, hsa-let-7d-5p, hsa-let-7e-5p, hsa-let-7f-5p, hsa-let-7g-5p, hsa-miR-144-3p and hsa-miR-26a-5p) were dramatically upregulated in H1975 and A549 cells after transfection with si-LINC00665-2 (Figure A1), as well as the protein expression of COL11A1 was significantly reduced (Figure 8e). The phenotypes of transfection with COL11A1 alone into H1975 and A549 cells were evaluated using CCK-8, transwell and TUNEL assays. After transfection with COL11A1 alone into H1975 and A549 cells, cell proliferation (Figure A2a) and migration (Figure A2b) were significantly accelerated compared with the control group. However, cell apoptosis had no obvious difference in the two groups (Figure A2c). Furthermore, si-LINC00665-2 and COL11A1 overexpressed plasmids were co-transfected into H1975 and A549 cells. si-LINC00665-2 transfection induced the inhibition of growth (Figure 8f) and migration (Figure 8g), and apoptosis (Figure 8h) was reversed by the overexpression of COL11A1, suggesting that the LINC00665-2/COL11A1 axis may contribute to the carcinogenesis of LUAD.

## 4 Discussion

In our study, a regulatory mechanism LINC00665-2/miRNAs/COL11A1 axis was identified to facilitate the tumorigenesis of LUAD. COL11A1 as a downstream gene target of LINC00665-2 and miRNAs was significantly elevated in LUAD tissues and served as a prognostic and diagnostic indicator. In addition, COL11A1 was positively correlated with multiple immune cells enrichment, suggesting that COL11A1 may be a prospective therapeutic target to enhance the efficacy of immunotherapy in LUAD.

Human pan-cancer studies reveal that COL11A1 expression is upregulated in approximately 14 cancer types and mediates inflammation and epithelial-mesenchymal transition phenotype in the tumor microenvironment that deteriorates cancer invasion and metastasis [2,44], reflecting that COL11A1 is a pivotal molecule to modulate cancer aggressiveness. Tu et al. demonstrated that COL11A1 is upregulated in NSCLC tissues and cell lines, and knockdown of COL11A1 inhibits colony formation ability and accelerates cell apoptosis in vitro experimental measurements [7]. Sun et al. proposed that COL11A1 is a posttranscriptional gene target of miR-144-3p that suppresses proliferation, migration and invasion of LUAD cells, suggesting that miR-144-3p-mediated COL11A1 inhibition may be an anticancer strategy for LUAD [45]. Our results suggested that COL11A1 might be controlled by multiple miRNAs via the upstream regulator LINC00665. LINC00665 knockdown enhanced nine miRNAs expression, as well as repression of COL11A1. A novel molecular mechanism axis lncRNA-LINC00665/miRNAs/COL11A1 is implicated in the progression of LUAD.

Previous studies indicate that the growth, invasion and metastasis of lung cancer have interacted with immune cells enrichment in the tumor microenvironment that may facilitate tumor cell escape and an unfavorable factor of antineoplastic immunotherapy [46,47]. T and B cells are the uppermost tumor-infiltrating immune cells in the majority of LUAD patients, and Th2, Th17 and Treg are correlated with unfavorable prognosis and metastasis of lung cancer [48]. Interestingly, B cell enrichment is markedly enhanced in human lung cancer compared with surrounding tissue or in distant nontumor tissues [46,49]. In several cancer types, COL11A1 correlates with multiple immune cells infiltration, such as macrophage, neutrophil, Th2 and dendritic cells in pancreatic adenocarcinoma [50,51], T cells, macrophages, neutrophils and dendritic cells in colon adenocarcinoma [2]. Herein, COL11A1 was significantly positively correlated with 10 immune cell enrichment in LUAD tissues, suggesting that COL11A1-mediated immune escape may partially account for the tumorigenesis of LUAD.

The other major finding was that lncRNA LINC00665 might be a regulator of COL11A1. *In vitro* experiments revealed that knockdown of LINC00665 downregulated COL11A1 protein expression in H1975 and A549 cells. Inhibition of LINC00665 abrogated the malignant phenotypes of H1975 and A549 cells. However, overexpression of COL11A1 neutralized the antineoplastic activities of si-LINC00665. Several studies have corroborate that LINC00665 expedites malignant progression and chemotherapy resistance of NSCLC [25,52,53].

In conclusion, bioinformatics analysis of the TCGA database and functional identification validated the oncogenic role of LINC00665 and COL11A1 in LUAD. Our findings provided a new insight into LINC00665 as a ceRNA sponging multiple miRNAs to modulate COL11A1 expression in LUAD, suggesting that LINC00665/miRNAs/COL11A1 axis may contribute to the pathogenesis of LUAD.

## Supplementary Material

Supplementary Table
